# Dilemmas in a pregnant woman with myelofibrosis secondary to signet ring adenocarcinoma: a case report

**DOI:** 10.1186/s12885-017-3666-x

**Published:** 2017-10-11

**Authors:** Pujun Guan, Zihang Chen, Li Zhang, Ling Pan

**Affiliations:** 1 Department of Hematology, West China Hospital, Sichuan University, No. 37 Guo-Xue Xiang, Chengdu, Sichuan 610041 China; 2 Department of Radiology, Huaxi Magnetic Resonance Research Centre (HMRRC), West China Hospital, Sichuan University, No. 37 Guo-Xue Xiang, Chengdu, Sichuan 610041 China; 3 Department of Pathology, West China Hospital, Sichuan University, No. 37 Guo-Xue Xiang, Chengdu, Sichuan 610041 China

**Keywords:** Myelofibrosis, Pregnancy, Gastric cancer, Signet ring adenocarcinoma

## Abstract

**Background:**

We describe the first reported case of myelofibrosis as an extremely rare complication of gastric cancer during pregnancy; the clinical diagnosis and treatment of which is highly challenging due to nonspecific symptoms coupled with the conflicting needs of immediate disease control and continuation of pregnancy.

**Case presentation:**

We report a 36-year-old pregnant woman who presented with cytopenia, fatigue, vomiting, and diarrhea for 20 days on the background of newly diagnosed myelofibrosis secondary to gastric signet ring adenocarcinoma. She accepted palliative care and died several months after the delivery of a healthy newborn.

**Conclusion:**

Signet ring gastric adenocarcinoma is an unusual cause of myelofibrosis during pregnancy. Treatment remains a great challenge as clinicians have to consider the needs of immediate treatment against fetal well-being while taking into account patient preference and fetus rights.

## Background

Myelofibrosis (MF) is a rare disease that can result from a multitude of reactive and neoplastic disorders. Secondary MF is commonly mistaken to be primary MF because the severe hematopoietic features may mask symptoms caused by the underlying primary disease(s) [1, 2]. The diagnoses and treatment of secondary MF during pregnancy are further complicated by a series of clinical dilemmas. We describe a late pregnant woman with MF secondary to metastatic bone marrow infiltration by signet ring adenocarcinoma (SRC) of the stomach. She died several months later while her newborn was safe and healthy.

## Case presentation

**Fig. 1:**
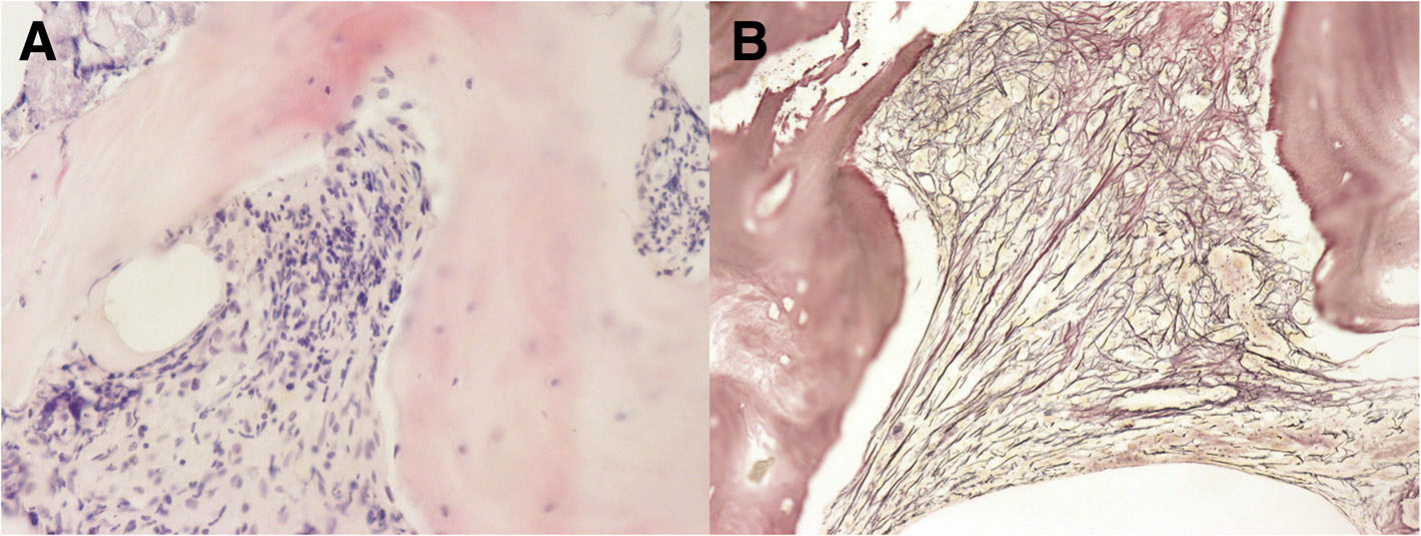
Bone marrow biopsy before delivery. **a** HE (200×). **b** Fibrosis ++ ~ +++. Foot-Menard Stain (200×)

The patient was a 36-year-old G2P1 patient at 28 weeks’ gestation whose chief complaint was fatigue for more than 20 days accompanied by vomiting, diarrhea, and cough with sputum for 10 days and right limb weakness for more than 3 days. She had a history of cesarean section in 2004 and pelvic fracture in 2006. The physical examination revealed significant ecchymosis in the right inguinal region and mild weakness of the right extremities (muscle strength grade 3/5) and normal muscle tone. Right Babinski sign was positive. Routine blood tests revealed thrombocytopenia (platelet count: 6 × 10^9/L; reference range100–300 × 10^9/L), anemia (hemoglobin: 62 g/L; reference range115-150 g/L), and leukocytosis (white blood cell count: 24.37 × 10^9/L; reference range 3.5–9.5 × 10^9/L) with 10% nucleus left shift. Fecal occult blood test was positive. A peripheral blood smear revealed increased red cell distribution width with basophilic stippling. Bone marrow aspiration was not successful due to dry tap while bone marrow biopsy showed grade 2 to 3 reticular fibrosis (Fig. 1). No JAK2 (Janus kinase 2) mutations and cytogenetic abnormalities were detected. Elevated levels of alkaline phosphatase (578 U/L) and lactate dehydrogenase (507 U/L) were detected along with a gradual one-month increase in tumor marker CA-125 (52.26 U/ml to 272.00 U/ml; reference range < 35 U/ml). Thyroid function test and immunophenotyping revealed slight hypothyroidism and suppression of cellular immunity respectively. Low-dose computed tomography (CT) scan showed low density small areas in the left insular lobe and besides left lateral ventricle angle, patchy areas with uneven density in pelvis and spine, splenomegaly with some infarction and enlarged lymph nodes around the stomach, and a little bit of the perioancreatic fat.

**Fig. 2:**
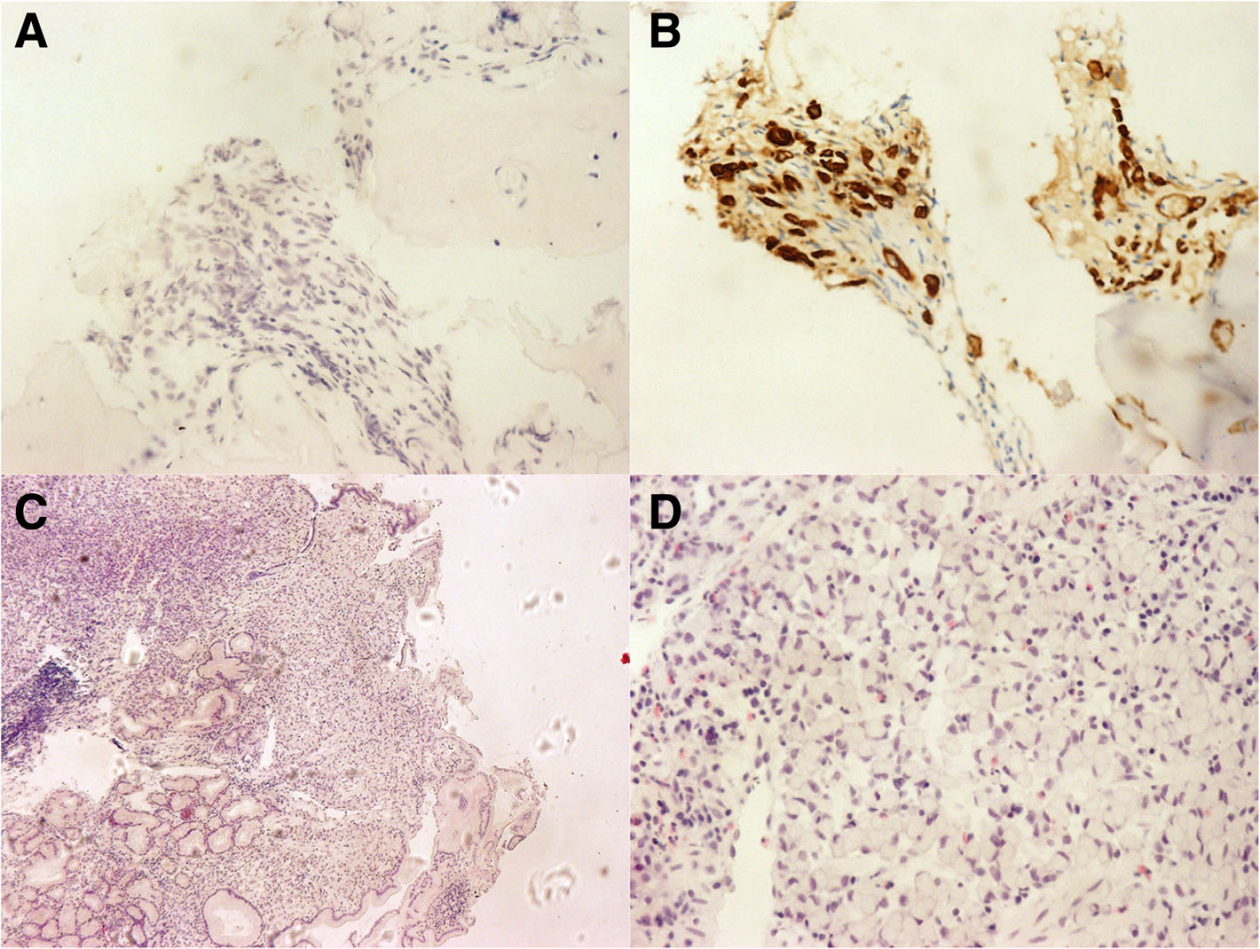
**a** & **b** Bone marrow biopsy after delivery. **a** HE (200×). **b** The tumor cells are CK pan positive (brown region, CK pan: a broad spectrum marker of epithelial cells; tumors which are originated from epithelium should be positive). (200×). **c** & **d** Gastric Mucosa Biopsy. **c** Signet ring cell infiltrated into gastric mucosa. HE (40×). **d** Signet ring cell. HE (400×)

The patient was given supportive care to continue pregnancy to 34 weeks. Three weeks later (the 31^st^ week), the patient complained of hematemesis accompanied by unbearable abdominal pain and then a cesarean section was operated. After that, positron emission tomography-computed tomography (PET-CT) with ^18^F–FDG revealed thickening of the gastric wall with accompanying increased uptake of glucose in the stomach and skeletal bones. Finally, a gastric biopsy was performed and the patient was diagnosed with SRC (Fig. 2). A repeat bone marrow biopsy revealed the presence of tumor metastasis and confirmed the diagnosis of MF secondary to bone marrow infiltration. The patient accepted palliative care and died several months later.

## Discussion

Pregnancy-associated cancer is a rare condition, with an estimated incidence of 1:10^6^ to 1:10^3^ pregnancies, depending on the type of cancer [3]. Kaoru Sakamoto et al. reported only 37 cases of pregnancy-associated gastric cancer from 1988 to 2007 in Japan; with SRC accounting for approximately 10% of cases [4]. We herein present the first reported case of MF secondary to SRC during pregnancy.

Primary MF is typically diagnosed in patients in their fifth or sixth decades and shows a significant male predominance [5]. This 36-year old pregnant patient with acute MF did not present with the cardinal signs of MF, i.e., her spleen was not palpable (slight splenomegaly on CT) and JAK2 mutational status was normal, thereby ruling out primary MF. And it is noteworthy that non-specific clinical features of gastric cancer can be masked by pregnancy and easily ignored. Based on the patient’s symptoms (vomiting and diarrhea) and an abnormal stool guaiac test, we then screened for infectious and metabolic causes to no avail but instead detected elevated levels of several tumor markers. The tumor marker CA-125 was monitored weekly and found to be increased from 52.26 U/ml to 272.00 U/ml within a month. The most likely diagnosis was therefore MF secondary to a gastrointestinal tumor.

Thus, the CT scan for brain, chest, abdomen and pelvis was performed to screen for whether distant metastases existed. However, pregnancy is a relative contraindication to CT scans [6]. This case demonstrates the classic maternal-fetal conflict. As physicians, we should balance the interests of the mother and her fetus while determining the management strategy. A literature search reviewed that the average uterine/fetal dose for chest CT (0.17 mGy) and abdominal-pelvic CT (18-25 mGy) are relatively low (less than 200-500 mGy) and are associated with an acceptable risk of adverse radiobiological events, although there is not a threshold dose for no injury [3]. A lower-dose abdominal CT protocol was applied; the CT dose index was 8.32 mGy (average of our center is 14 mGy) which might be lesser impact on the fetus well-being.

However, endoscopy was not performed accordingly for this case during pregnancy. The previous study indicated that gastroscopy is innocuous and should be done when clinically required during pregnancy, unless patients have obstetric complications such as placental abruption, imminent delivery, ruptured membranes, or eclampsia [3, 7]. This patient had thrombocytopenia (platelet count persistently below 20 × 10^9/L), which is a relative contraindication to gastroscopy. Additionally, the patient refused to undergo gastroscopy without sedation because of pain intolerance. Painless gastroscopy under sedation, on the other hand, would place endanger both the patient and her fetus due to maternal/fetal hypoxia [8]. In order to protect the pregnant woman and her fetus, endoscopy was delayed for 3 weeks after parturition.

Supportive therapy was finally applied, while abortion was not practiced for this case. Although the decision to continue on with the pregnancy was a tough one as the patient’s condition was deteriorating, societal norms and expectations shaped by cultural and religion play an essential role in the decision-making process [9, 10]. Eventually, we managed the patient with supportive therapies such as hemostasis, transfusion and nutritional support up to the point of delivery and achieved an acceptable outcome.

## Conclusion

In general, we present a rare cause of MF secondary to gastric SCR in a pregnant woman and provide both clinical restrictions and ethical dilemmas surrounding the management of this patient. The diagnosis may be delayed as mild gastrointestinal symptoms are common during pregnancy, while early detection of gastric cancer is critical to ensure better outcomes.

## Abbreviations

^18^F–FDG: 2-Deoxy-2-fluoro-D-glucose
CT: Computed tomography
*JAK2*: *Janus kinase 2* gene
MF: Myelofibrosis
PET-CT: Positron emission tomography–computed tomography
SRC: Signet ring adenocarcinoma
